# Predictors of new atherosclerotic carotid plaque development in patients with rheumatoid arthritis: a longitudinal study

**DOI:** 10.1186/ar3757

**Published:** 2012-03-05

**Authors:** Evangelia Zampeli, Athanase Protogerou, Kimon Stamatelopoulos, Kalliopi Fragiadaki, Christina G Katsiari, Katerina Kyrkou, Christos M Papamichael, Myron Mavrikakis, Peter Nightingale, George D Kitas, Petros P Sfikakis

**Affiliations:** 1 First Department of Propaedeutic and Internal Medicine, Laikon Hospital, Athens University Medical School, Ag Thoma, 17, GR-11527 Athens, Greece; 2 Vascular Laboratory, Department of Clinical Therapeutics, Alexandra Hospital, Athens University Medical School, V Sofias and Lourou 1, GR-11528, Athens, Greece; 3 Wellcome Trust Clinical Research Facility, University Hospital Birmingham NHS Foundation Trust, Birmingham, Queen Elizabeth Hospital, Queen Elizabeth Medical Centre, Birmingham, B15 2TH, UK; 4 The Dudley Group of Hospitals NHS Foundation Trust, Dudley, and Arthritis Research Campaign Epidemiology Unit, University of Manchester, Manchester, West Midlands, DY1 2HQ, UK

## Abstract

**Introduction:**

Rheumatoid arthritis (RA) is associated with increased cardiovascular morbidity and mortality attributed to both classical risk factors and chronic inflammation. We assessed longitudinally the factors associated with new carotid plaques in nondiabetic RA patients and apparently healthy individuals.

**Methods:**

In our present prospective observational study, carotid plaques were identified by ultrasonography at baseline and follow-up end, separated by an average of 3.6 ± 0.2 years, in 64 patients (mean age 59.2 ± 12.0 and disease duration at baseline 7.8 ± 6.2 years, 83% women, clinical and laboratory evaluation every 3 to 6 months). In a substudy, 35 of the patients were matched 1:1 for traditional cardiovascular risk factors with 'healthy' controls and were studied in parallel.

**Results:**

New atherosclerotic plaques formed in 30% of patients (first plaque in 9%) who were significantly older than the remaining patients. Tobacco use, blood pressure, body mass index, average cumulative low-density lipoprotein, high-sensitivity C-reactive protein, erythrocyte sedimentation rate level, RA stage, functional class, disease duration and treatment modalities during follow-up did not differ significantly between subgroups after application of the Bonferroni correction. RA was in clinical remission, on average, for approximately 70% of the follow-up time and was not different between subgroups. Multivariate analysis including all the above parameters revealed that age (*P *= 0.006), smoking (*P *= 0.009) and duration of low-dose corticosteroid use (*P *= 0.016) associated independently with new plaque formation. RA patients displayed similar numbers of newly formed carotid plaques to the tightly matched for traditional cardiovascular risk factors 'healthy' controls, although more patients than controls had carotid plaques at baseline.

**Conclusions:**

Formation of new atherosclerotic plaques in this small cohort of patients with well-controlled RA depended mainly on traditional cardiovascular risk factors and corticosteroid use, whereas an adverse effect of residual systemic inflammation was not readily detectable.

## Introduction

Rheumatoid arthritis (RA) is an independent risk factor for cardiovascular disease (CVD) that is associated with at least a 1.5-fold increased risk for a fatal coronary event compared to the general population [1]. This is based predominantly on data derived from epidemiological studies conducted during the past two or three decades, which included many RA patients diagnosed and managed before the era of aggressive, target-driven therapy with disease-modifying antirheumatic drugs (DMARDs), including biologic agents [2]. Complementary data deriving from cross-sectional studies have shown that RA patients have evidence of advanced preclinical carotid atherosclerosis compared to healthy controls [3] to a magnitude similar to that observed in patients with diabetes mellitus (DM) [4]. High-resolution B-mode ultrasonography of the carotid artery provides a noninvasive, valid and reproducible method for identifying atherosclerotic plaques, which reflect prevalent, clinical or preclinical CVD and may represent predictors of future CVD events [5]. Notably, in RA patients without traditional CVD risk factors or events, an increased intima-media thickness (IMT) of the common carotid artery and evidence of focal plaques were each predictive of incident CVD events [6,7].

There is an increased burden of traditional CVD risk factors in RA patients [8], including hypertension [9], dyslipidaemia [10], smoking [11], insulin resistance [12], obesity and/or altered body composition [13,14] and physical inactivity [15], but this accounts only partly for the excess CVD mortality [16]. Because inflammation plays a major role in the atherosclerotic process [17], interest has focused on the impact of the systemic inflammatory burden and chronic activation of the immune system [18] present in RA [19,20]. Given the paucity of prospective data, the extent to which traditional CVD risk factors and RA-related parameters (inflammatory burden, activity and/or remission and treatment modalities) interact and/or contribute to atherosclerosis acceleration remains inconclusive [21]. According to a recent study, both traditional CVD risk factors and markers of RA severity at baseline contribute to models predicting cardiovascular (CV) events in the subsequent 22 months [22].

In the present study, we assessed (1) the factors associated with the formation of at least one new carotid plaque per subject during the follow-up period in nondiabetic RA patients and (2) whether the number of newly formed plaques in these patients differs from the respective number observed in non-RA subjects carefully matched for traditional CVD risk factors.

## Materials and methods

### Study population and design

To address the first objective mentioned above, we used a unique prospective cohort comprising RA patients who met the American College of Rheumatology classification criteria [23] and participated in this observational study. This convenience sample was composed of RA patients who initially participated in the cross-sectional study (from 2006 to 2007) [4], who were asked consecutively to provide informed consent and were willing and available to be reassessed after 3 years. Exclusion criteria at baseline were the presence of CVD (based on positive medical history, and/or clinical examination, and/or ECG abnormalities), DM, uncontrolled hypothyroidism, renal impairment and malignancy or treatment with nonsteroidal anti-inflammatory drugs (NSAIDs). Subclinical carotid atherosclerosis was evaluated at baseline and during the fourth year after baseline (2009 to 2010). To address the second objective mentioned above, we used a cohort of apparently 'healthy' control subjects. RA patients were matched 1:1 with controls for all the traditional CV risk factors (age ± 1 year, sex, smoking habits, body mass index (BMI) ± 3 kg/m^2^, hypertension and/or antihypertensive drug treatment and dyslipidaemia and/or statin treatment) present not only at baseline but also at the end of follow-up. Control subjects were recruited among hospital personnel or patients' 'buddies' and underwent the same evaluations at baseline and follow-up end. Matching was possible for 35 RA-control pairs.

The study was subject to Institutional Body Review, and all subjects provided their informed consent according to the Declaration of Helsinki.

### Evaluation and management of RA patients

Clinical evaluation and laboratory tests (cholesterol, low-density lipoprotein (LDL), erythrocyte sedimentation rate (ESR) and high-sensitivity C-reactive protein (hs-CRP)) were performed at baseline and at every regular follow-up visit thereafter (every 3 to 6 months), as well as at any time a patient experienced a disease flare. Rheumatoid factor (RF), functional status (class I, II, III or IV) [24] and progression of RA (early, moderate, severe or terminal stage) [25] were assessed at baseline for each patient.

At each visit, three blood pressure (BP) measurements were taken (Omron 705CP; Omron Healthcare, Inc, Lake Forest, IL, USA) in the right upper limb after 5 minutes of rest with the subject seated and the arm supported at heart level, and the average BP was used. In the absence of antihypertensive treatment, "hypertension" was defined as the presence of systolic BP (SBP) ≥ 140 mmHg and/or diastolic BP (DBP) ≥ 90 mmHg [11]. Similarly, in the absence of lipid-lowering treatment, dyslipidaemia was defined as LDL ≥ 160 mg/dl [26]. Periods of uncontrolled BP and periods of dyslipidaemia (in months) were recorded and summed for each patient using these cutoffs. Family history of coronary artery disease (CAD) was defined as positive if a cardiac event (fatal or nonfatal myocardial infarction or coronary angioplasty and/or coronary artery bypass surgery) had occurred before the age of 55 years in male first-degree relatives and before the age of 65 years in female first-degree relatives.

Periods of clinical remission of RA during follow-up were recorded (in months) and summed for each patient. For this purpose, remission was not defined by the Disease Activity Score in 28 joints, often used in clinical trials, which applies for a given day (joint counts) and the previous week (Visual Analogue Scale), but on the basis of at least five of the following six criteria being present for at least 2 months: (1) duration of morning stiffness < 15 minutes, (2) no fatigue, (3) no joint pain, (4) no joint tenderness and/or pain on motion, (5) no soft-tissue swelling and (6) ESR < 30 mm/hour for females or < 20 mm/hour for males [27]. Patients were treated according to guidelines for optimal RA control (that is, disease remission) while avoiding administration of NSAIDs [28].

### Assessment of carotid plaque

The presence of atherosclerotic plaques in the carotid arteries was determined using a 14.0-MHz multifrequency linear array probe attached to a high-resolution ultrasound machine (Vivid 7 Pro; GE Healthcare, Little Chalfont, Buckinghamshire, UK), based on the international recommendations [29]. In each subject, the following six sites (three paired left and right segments) were used for the investigation of atherosclerotic plaques: (1) at the common carotid artery, defined as the segment 1 cm proximal to carotid dilation; (2) at the carotid bulb, defined as the segment between the carotid dilation and carotid flow divider; and (3) at the internal carotid artery, defined as a 1-cm-long arterial segment distal to the flow divider. All segments were identified in the transverse and/or longitudinal planes and scanned from multiple angles to optimize the detection of unobstructive plaques. Atherosclerotic plaques were defined at baseline or at follow-up end as focal structures at the far wall that encroached into the arterial lumen at least 0.5 mm or 50% of the surrounding IMT value or that demonstrated a thickness > 1.5 mm measured on a digitally stored image [29]. Baseline images were reevaluated offline using Artery Measurement Software [30] concurrent with follow-up images to avoid interpretation bias.

### Statistical analysis

Statistical analysis was performed using SPSS version 18.0 software (SPSS, Inc, Chicago, IL, USA), and significance was defined as *P *< 0.05 throughout. Variables were tested for normality using the Kolmogorov-Smirnov test. For each patient, the average of values obtained during the follow-up period (at least seven separate values of SBP and DBP, LDL, hs-CRP and ESR), the respective areas under the curve (AUCs) and the HeartScore [31] were calculated. The results are presented as means ± SD or as percentages, and non-normally distributed continuous variables are presented as median values with lower and upper quartiles.

For the first objective, patients who developed new plaques during the follow-up period were compared with those who did not by using an independent sample *t*-test, analysis of variance and the Mann-Whitney *U *test (comparison of continuous variables as appropriate), as well as the χ^2 ^or Kendall's tau-b test (comparison of noncontinuous variables as appropriate). To find the independent predictors of new plaque formation in the totality of the RA sample, we applied binary logistic regression analysis using both enter and backward methods. The possible presence of interaction between systemic inflammation and traditional risk factors was tested by introducing into the multivariate model the product of hs-CRP with each classical CV risk factor. For the second objective, the characteristics of the matched populations at baseline and at the end of the follow-up period were compared using a paired *t*-test and Wilcoxon test. To compare the progression of the number of plaques within each group, we used a paired *t*-test and Wilcoxon test.

## Results

### Characteristics of patients

As reported elsewhere [4], we cross-sectionally investigated 84 nondiabetic RA patients (mean age 59.2 ± 12.1 years, 70% females). For 64 of them (age 59.2 ± 12.0 years at baseline, 83% females), a prospective evaluation every 3 to 6 months was possible for 40.8 ± 3.0 months (mean ± SD). Mean disease duration at baseline was 7.8 ± 6.2 years, and 65% were RF-positive. Thirty patients (47%) had at least one carotid plaque at baseline. Hypertension, dyslipidaemia, current tobacco use and family history of CAD were present in 46%, 31%, 34% and 21% of patients, respectively. Throughout the follow-up period patients were in remission for a median of 34.1 months (20.7 to 38.4). Cumulative ESR and hs-CRP values for the entire follow-up period were, on average, 26.7 ± 13.4 mm/hour and 0.5 mg/dl (0.29 to 1.19), respectively. Myocardial infarction occurred in one male patient, and one additional male patient and one female patient underwent carotid stenting during follow-up. All three patients remained in the study, and new plaque formation in the contralateral artery was assessed.

### New carotid atherosclerotic plaque formation

At follow-up end, 30% of the patients had developed at least one new carotid plaque. First plaque was noted in six (9%) of them. RA-related parameters, including duration of disease remission and treatment modalities implemented, as well as traditional CV risk factors, statin use and antihypertensive treatment in subgroups of patients stratified by the presence of new carotid plaque, are presented in Table 1. Although 13 of 28 comparisons reached statistical significance, including smoking, months of treatment with corticosteroids (between 2.5 and 10 mg prednisolone/day), cumulative number of traditional risk factors and carotid plaques at baseline (all *P *< 0.02), only age was significantly different between those RA patients who developed new plaque and those who did not after we applied the Bonferroni correction for multiple comparisons (*P *= 0.008).

**Table 1:** Rheumatoid arthritis-related parameters and traditional cardiovascular risk factors in subgroups of nondiabetic rheumatoid arthritis patients stratified by the presence of at least one new carotid plaque at follow-up end^a^

	New carotid plaque formation during follow-up	
	
Parameters	Yes	No
Patient demographics		
Total number of patients	19	45
Age at follow-up end (years)^b^	70.6 ± 6.9	59.2 ± 12.1
Sex, *n *(%)	14 women (73.7) 5 men (26.3)	39 women (86.7) 6 men (13.3)
Months of follow-up (months)	40.6 ± 3.0	40.9 ± 3.0
RA-related parameters		
Duration of disease at follow-up end (years)	9.4 ± 3.9	11.9 ± 6.9
Functional class of disease, *n *(%)		
Class I	12 (63.2)	18 (40.0)
Class II	6 (31.6)	17 (37.8)
Class III	1 (5.3)	9 (20.0)
Class IV	0 (0)	1 (2.2)
Stage of disease, *n *(%)		
Early	6 (31.6)	11 (24.4)
Moderate	12 (63.2)	19 (42.2)
Severe	1 (5.3)	14 (31.1)
Terminal	0 (0)	1 (2.2)
RF-positive, *n *(%)	13 (68.4)	29 (64.4)
hs-CRP AUC during follow-up (mg/dl × months)	16.6 (12.4, 57.5)	23.8 (12.3, 45.2)
ESR AUC during follow-up (mm/hour × months)	1,121.1 ± 627.6	1,157.0 ± 537.6
Disease-related treatments (months during follow-up)		
Nonbiologic DMARDs	38.4 (35.1, 41.4)	40.0 (32.6, 42.5)
Biologic agents	0.0 (0.0, 39.3)	31.6 (0.0, 41.4)
Corticosteroids	40.5 (35.2, 42.3)	33.2 (3.7, 41.7)
Remission during follow-up (months)	36.2 (32.4, 39.1)	31.4 (11.3, 37.3)
Remission during follow-up (% of follow-up time)	90.5 (85.0, 92.9)	84.6 (27.8, 92.6)
Traditional cardiovascular risk factors		
Hypertension at baseline, *n *(%)	12 (66.7)	17 (37.8)
SBP AUC during follow-up (mmHg × months)	5,791.1 ± 634.3	5,416.7 ± 599.6
DBP AUC during follow-up (mmHg × months)	3,491.4 ± 306.5	3,333.9 ± 323.9
Antihypertensive treatment (% follow-up time)	2.3 (0, 2.4)	0 (0, 2.3)
LDL ≥ 160 mg/dl and/or under statin treatment at baseline, *n *(%)	8 (42.1)	12 (26.7)
LDL AUC during follow-up (mg/dl × months)	5,528.9 ± 1294.1	5,457.7 ± 1377.9
Statin use during follow-up (% follow-up time)	0.0 (0.0, 29.0)	0.0 (0.0, 0.0)
Smoking at end of follow-up, *n *(%)	7 (36.8)	14 (31.1)
Total years of smoking	30 (0.0, 40.0)	0.0 (0.0, 29.0)
BMI (kg/m^2^)		
BMI at baseline	26.1 ± 3.5	25.9 ± 3.7
BMI at follow-up end	26.0 ± 3.8	26.3 ± 4.4
Positive family history of CAD, *n *(%)	7 (36.8)	8 (17.8)
Number of CV risk factors, *n *(%)		
0	0 (0)	13 (28.9)
1	9 (47.4)	17 (37.8)
2	7 (36.8)	12 (26.7)
3	3 (15.8)	3 (6.7)
Carotid plaques at baseline, *n *(%)	14 (73.7)	16 (35.6)

^a^AUC: area under the curve, BMI: body mass index, CAD: coronary artery disease, CV: cardiovascular, DBP: diastolic blood pressure, ESR: erythrocyte sedimentation rate, hs-CRP: high-sensitivity C-reactive protein, LDL: low-density lipoprotein, RA: rheumatoid arthritis, SBP: systolic blood pressure. Data are number (%), mean ± SD or median (lower and upper quartiles). ^b^Significant difference between subgroups after Bonferroni correction for multiple comparisons (*P *= 0.0084). All other comparisons did not reach significance.

Thirteen of the sixty-four RA patients were free of traditional CVD factors: hypertension, dyslipidaemia and smoking. As expected, they were significantly younger (47.9 ± 14.3 years) than the remaining patients. None of them developed new carotid plaque during follow-up, although they had disease duration at baseline comparable with the remaining patients (data not shown).

### Factors associated with atherosclerotic plaque formation

We searched for parameters predictive of new carotid plaque formation by binary logistic regression analysis. Because of the limited number of RA patients who developed a new plaque, we evaluated the combined effect of traditional CV risk by introducing into the models (enter method) HeartScore together with (one at a time) RA-related parameters (RF positivity, functional class and stage of RA at baseline and biologic and nonbiologic DMARDs). HeartScore at baseline (B = 0.256, *P *= 0.011) and months of corticosteroid use during follow-up (B = 0.069, *P *= 0.013) were independent predictors of new plaque formation (*r*^2 ^= 0.345). Because the subgroup of patients with new atherosclerotic plaque formation was older by 11 years than the subgroup without, however, traditional risk factors (for example, longer smoking duration) may have already had an opportunity to 'take a toll' on the arteries before RA clinically manifested. To further examine determinants of new carotid plaque formation, we matched patients from these subgroups 1:1 for age (± 3 years) and sex. Among 15 patient pairs, we found that the only determinant of new plaque formation was corticosteroid use duration (*P *= 0.044).

We then investigated the contribution of individual CV risk factors by multivariate analysis using both enter and backward methods (Table 2). First, traditional CV risk factors (age, sex, smoking, BP and BMI) were entered into the multivariate model (model 1). Only age and total years of smoking independently predicted new carotid plaque formation. Second, only parameters related to RA disease status (RF positivity, functional class and stage of RA at baseline, percentage in remission during follow-up, and hs-CRP) were entered into the model (model 2, data not shown in Table 2). None of these parameters predicted new carotid plaque formation. Third, in model 3, only treatment modalities (corticosteroids, biologic and nonbiologic DMARDs) were entered into the model. In the subgroup of patients who developed at least one new carotid plaque during follow-up, biologic agents were given for a median of 0.0 (0.0 to 39.3) months and comprised mainly anti-TNF agents (0.0 to 28.5 months). Cytotoxic T-lymphocyte antigen 4 immunoglobulin, IL-6R inhibitor, anakinra and rituximab were also used in isolated patients. It was found that duration of corticosteroid use was positively associated with new carotid plaque formation. In contrast, duration of biologic DMARD use was a negative independent predictor of new carotid plaque formation. Therefore, only the independent predictors from models 1, 2 and 3 (that is, age, smoking, corticosteroids and biologic agents) were entered into a final model. Age, smoking and corticosteroids remained significant and independent predictors of new plaque formation. Notably, no interaction between classical CV risk factors and hs-CRP was found in either model 1 or the final model. No significant alterations were observed after further adjustment of the final model for family history for CAD, baseline plaques or CV risk reduction drugs (statins and antihypertensive agents).

**Table 2:** Multivariate models predicting new carotid plaque formation in nondiabetic patients with rheumatoid arthritis over 3.6 ± 0.2 years^a^

	New carotid plaque formation during follow-up (yes vs no)		
		
Parameters	β coefficient	*P *value	Odds ratio (95% CI)
Model 1: traditional CV risk factors (*r*^2 ^= 0.0453)			
Age at follow-up end (years)	0.146	0.004	5.752^b ^(1.770 to 18.694)
Sex	0.038	0.970	0.963^b ^(0.134 to 6.927)
Total years of smoking (at follow-up end)	0.051	0.023	2.628^b ^(1.145 to 6.029)
SBP AUC (mmHg × months)	0.001	0.346	1.495^b ^(0.648 to 3.449)
LDL AUC (mg/dl × months)	0.000	0.126	0.507^b ^(0.212 to 1.211)
BMI (at follow-up end)	0.088	0.356	1.445^b ^(0.661 to 3.160)
Model 3 RA treatment (months of treatment during follow-up) (*r*^2 ^= 0.248)			
Corticosteroids	0.067	0.016	3.058 (1.231 to 7.593)
Biologic agents	-0.035	0.046	0.502 (0.255 to 0.987)
Nonbiologic DMARDs	-0.017	0.477	0.802 (0.437 to 1.473)
Final model (*r*^2 ^= 0.559)^c^			
Age at follow-up end (years)	0.136	0.006	5.098 (1.599 to 16.250)
Total years of smoking (at follow-up end)	0.055	0.009	2.875 (1.305 to 6.335)
Corticosteroids (months during follow-up)	0.086	0.016	4.170 (1.299 to 13.385)
Biologic agents (months during follow-up)	-0.033	0.118	0.520 (0.230 to 1.180)

^a^AUC: area under the curve, BMI: body mass index, CV: cardiovascular, DMARDs: disease modifying antirheumatic drugs, LDL: low-density lipoprotein, RA: rheumatoid arthritis, SBP: systolic blood pressure. ^c^Only the variables from models 1 to 3 that predicted new plaque formation were used in the final model. Model 2 is described in the text only. ^b^The odds ratio for the continuous values was assessed for 1 SD increase and so is provided by exp(β*SD) (SD for age 12.0, for total years of smoking 19.15, for SBP (AUC) 629.10, for LDL (AUC) 1343.77, for BMI 4.25, for corticosteroid treatment (months during follow-up) 16.60, for biologic agents received (months during follow-up) 19.90, for nonbiologic DMARDs (months during follow-up) 13.20). Dependent variable 0 is subjects with no new plaque (*n *= 45), variable 1 is subjects with at least one plaque (*n *= 19) at the end of follow-up (binary logistic regression analysis using enter method is presented, and identical results in all models were obtained using the backward method).

### Comparable new carotid plaque formation in rheumatoid arthritis and matched controls

Thirty-five pairs of RA patients and non-RA subjects who were successfully matched 1:1 for all the traditional CV risk factors (sex, age, smoking, BMI, hypertension and dyslipidaemia) and CV risk reduction drugs, both at baseline and at follow-up end, were included in this analysis (Table 3). The RA subgroup was only in part representative of the entire cohort, because comparison with the remaining 29 patients showed significantly younger age and significantly less baseline carotid plaques. All other RA-related parameters and CV risk factors were not significantly different (data not shown). After adjustment for age, the difference in the proportion of patients with plaques at baseline (that is, 12 of 35 versus 18 of 29 (*P *= 0.044 before adjustment)) became nonsignificant (*P *= 0.325). These 35 RA patients were in remission, on average, for a median of 30.1 months (11.1 to 38.3), that is, 84.1% (26.3 to 92.4) of the follow-up period, whereas the average cumulative values throughout the follow-up for ESR and hs-CRP were 27.4 ± 13.8 mm/hour and 0.3 mg/dl (0.1 to 0.6), respectively. In total during follow-up, these patients had received 23.0 months (0.0 to 41.5) of treatment with biologic agents, 30.0 months (0.0 to 41.6) of methotrexate therapy and 37.3 months (15.1 to 42.7) of corticosteroid treatment (always < 10 mg prednisolone/day).

**Table 3:** Characteristics of 35 nondiabetic rheumatoid arthritis patients (29 women, 11 with hypertension, 10 with dyslipidaemia, 11 smokers) who were matched 1:1 with 'healthy' control subjects for sex, age (59.6 ± 11.4 years) and other traditional cardiovascular risk factors present both at baseline and at follow-up end^a^

Parameters	Matched 'healthy' control subjects	Subgroup of 35 RA patients
Number of patients	35	35
Follow-up (months)	40.4 ± 3.4	41.4 ± 3.3
hs-CRP (mg/dl)		
Baseline	0.1 (0.05, 0.2)	0.3 (0.2, 0.6)
Follow-up end	0.2 (0.08, 0.3)	0.4 (0.2, 0.8)
Traditional CV risk factors		
Hypertension (mmHg)		
SBP baseline	122.7 ± 21.0	127.0 ± 20.0
SBP follow-up end	123.6 ± 19.1	128.6 ± 13.9
DBP baseline	75.6 ± 12.0	76.9 ± 9.5
DBP follow-up end	75.9 ± 8.9	80.4 ± 7.5
Dyslipidaemia (mg/dl)		
LDL baseline	141.3 ± 42.6	136.4 ± 35.6
LDL follow-up end	128.3 ± 31.0	116.2 ± 34.0
Smoking		
Pack-years at baseline	0.0 (0.0, 35.0)	0.0 (0.0, 21.4)
Pack-years during follow-up	0.0 (0.0, 3.0)	0.0 (0.0, 1.5)
BMI (kg/m^2^)		
Baseline	25.3 ± 4.3	26.5 ± 4.0
Follow-up end	25.5 ± 4.1	26.9 ± 4.7

^a^BMI: body mass index, CV: cardiovascular, DBP: diastolic blood pressure, hs-CRP: high-sensitivity C-reactive protein, LDL: low-density lipoprotein, SBP: systolic blood pressure. Paired *t*-tests and Wilcoxon tests were used for all comparisons of the matched groups as appropriate. Variables are expressed as mean ± SD or as median (lower and upper quartiles). Comparisons between subgroups after Bonferroni correction did not reach significance.

**Figure 1:**
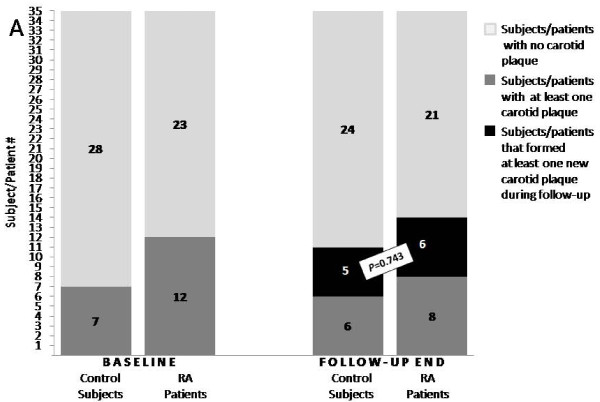
**Comparable new carotid plaque formation in rheumatoid arthritis patients and matched controls**. New carotid plaque formation during a mean (± SD) of 3.4 ± 0.3 years in 35 rheumatoid arthritis (RA) patients matched 1:1 with 35 'healthy' subjects for all traditional cardiovascular risk factors at baseline and at the end of follow-up. Although at baseline almost twice as many RA patients as controls presented with at least one carotid plaque, the number with new carotid atherosclerotic plaque formation was similar to that of controls (*P *= 0.743).

As shown in Figure 1, despite the fact that more patients had carotid plaques at baseline (34% versus 20%, albeit not reaching significance owing to sample size), only six RA patients developed at least one new plaque (two of them had no plaque at all at baseline) compared with five control subjects (four of them had no plaque at baseline) (relative risk = 1.20, 95% CI 0.35 to 4.27, *P *= 0.743). Collectively, the number of plaques per subject increased significantly in both groups during follow-up (from 0.26 ± 0.56 to 0.51 ± 0.88 plaques per subject in the control group, *P *< 0.001; from 0.51 ± 0.81 to 0.71 ± 1.0 plaques per subject in the RA group, *P *< 0.017). The change in plaques per subject between the groups was equal: 0.24 ± 0.66 versus 0.20 ± 0.47, respectively (*P *= 0.761).

## Discussion

In this prospective, longitudinal study, we examined factors leading to new carotid plaque formation in nondiabetic patients with RA without CVD at baseline. As recently reported, carotid atherosclerosis predicts future coronary events in RA, whereas those patients with carotid plaques, multiple CV risk factors (particularly DM or hypertension), many swollen joints and a high cumulative dose of glucocorticoids, as well as male RA patients, are at even higher risk [7]. We focused on new carotid plaque formation and not on IMT progression, although both biomarkers have been associated with CV events in RA and non-RA populations and may have additive predictive value [6,32]. Our decision to exclude IMT from the analysis was based on the fact that, though focal plaque is the direct manifestation of atherosclerosis, IMT is considered a surrogate not only of diffuse atherosclerosis but also of medial hypertrophy, unrelated to atherosclerosis, that is driven primarily by hypertension [32].

Our study produced three main novel findings which were consistently verified by different statistical approaches. First, in well-treated RA patients in clinical remission for more than two-thirds of the follow-up period, new carotid plaque formation was predominantly associated with traditional CVD risk factors. Second, of all RA-related parameters evaluated, only chronic corticosteroid use was independently associated with new carotid plaque formation. Third, over an average 3.5 years follow-up period, the number of new carotid plaques in the subgroup of RA patients and the tightly matched for traditional CVD risk factors non-RA subjects, was similar.

In the present study, a relatively small sample size was available, probably resulting in insufficient statistical power to detect small differences, especially at baseline, between patients with or without new plaque formation during follow-up. Another limitation is the fact that we focused solely on new plaque formation and did not assess the progression of preexisting plaques. Such assessments ideally require three-dimensional models that were not available to us, whereas the potential error that would have been introduced by the available two-dimensional models might have been too large.

Cross-sectional studies have shown that the severity of inflammatory burden in long-standing RA measured by CRP, as well as disease duration, are associated with increased preclinical atherosclerosis in patients without traditional CVD risk factors [33]. In the present prospective study, however, we did not observe any association between new carotid plaque formation and RA disease-related parameters, including CRP and ESR levels. We think that, in a prospective study, the influence of patients' characteristics at baseline is less important than those present during the follow-up period. The lack of such an association may be explained by the low residual inflammation present in our optimally treated patient cohort during the follow-up period as well as by the relatively high burden of traditional CV risk factors in the present cohort, which constituted a typical RA population. All patients were managed according to the latest approaches of treating to target (that is, disease remission) with very frequent follow-up, rapid escalation of disease-modifying therapy and unrestricted access to biologic agents (given in 52% of patients), thus resulting in clinical remission for more than two-thirds of the follow-up time. As most recently reported in another prospective study assessing longitudinal predictors of progression of carotid atherosclerosis in RA, inflammation was indeed a contributor. However, the probability of atherosclerosis progression was minimal up to an average CRP threshold of 12 mg/L, whereas, more importantly, the average CRP (median of cumulative average during study period of 3.1 mg/L) in nondiabetic RA patients was not associated with carotid atherosclerosis progression [34].

The finding that the formation of new carotid plaques in patients with well-controlled RA depends mainly on traditional CVD risk factors was reproduced in the comparative cohort study. In this comparative study, patients had higher BP than controls by almost 5 mmHg, yet they developed a similar number of new plaques over a 3.5-year period. Because the younger age of patients in the RA matched population versus the remaining patients could constitute a selection bias, part of the results may not be representative and thus cannot be extrapolated to other age groups.

The findings presented herein add credence to the current European Union League Against Rheumatism guidelines on CVD risk management in RA [1]. Because our present study was conducted before the publication of these guidelines, our patients did not receive aggressive CVD risk reduction management. Smoking, which has a higher prevalence in Greece than in other European Union countries [35], and age were the main factors defining the new carotid plaque formation in the present population, as expected in non-RA populations [36,37]. This finding clearly shows the need to optimally control classical CVD risk factors in RA patients, factors which are often neglected in everyday clinical practice. Of note, no interaction between inflammation and classical CVD risk factors and no synergy between them were found. Also, we did not find any interaction between age- and RA-related parameters (inflammation or drugs) regarding their effect on new plaque formation. However, since the age at onset of RA was greater in patients who developed new plaques than in those who did not (61.2 ± 7.8 versus 47.3 ± 12.7 years of age respectively, *P *< 0.001), it is possible that older arteries may also respond less well to inflammatory burden and/or steroids and hence the process of atherosclerosis may be accelerated.

In contrast to the known detrimental role of high-dose corticosteroids in CVD risk, until recently there was no clear evidence for the role of a chronic low daily dose of corticosteroids in patients with inflammatory arthritis [1,9,10]. In general, the net effect of corticosteroids on vascular function in chronic inflammatory disease is obscure [38]. As reported by Evans *et al.*, higher cumulative glucocorticoid dose does confer a higher risk of acute coronary syndromes in RA [7]. Even more recently, Giles *et al. *reported prospective data suggesting that subclinical carotid atherosclerosis progression in RA is potentially modified detrimentally by cumulative prednisone exposure [34]. Our results also suggest that low doses of corticosteroids influence significant new plaque formation in RA. It should be noted, however, that these are not randomised study-derived data and may thus represent a low level of proof. Moreover, a detrimental effect of corticosteroids is a confounding factor by indication, because it could reflect higher disease activity and may be associated with RA severity [39].

Although biologic therapies appear to decrease the risk of CVD events in RA patients [40], inconsistent data have been reported in several papers on the effects of biologics, primarily TNF blockers, on carotid atherosclerosis, depending on the treatment duration and clinical efficacy of treatment [41,42]. Whether the positive effect of these drugs is related to their anti-inflammatory actions, leading to a decreased risk of unstable plaques, rather than to an effect on the size of carotid IMT deserves further studies. In the initial multivariate analysis, we found a marginally significant beneficial effect of biologic DMARDs on the atherosclerotic process, but the significance was lost in the final model (Table 2). Other prospective observations suggest that subclinical carotid atherosclerosis progression in RA is potentially modified favourably by TNF inhibitors [34]. Taken together, these data imply that particular treatment modalities that induce disease remission, such as corticosteroids and TNF inhibitors, may be very important or even more important than disease remission *per se *as far as the subclinical atherosclerosis in RA is concerned.

## Conclusion

Formation of new atherosclerotic plaques in this small cohort of optimally treated patients with well-controlled RA depended on traditional CVD risk factors and to a lesser extent on corticosteroid use, whereas an adverse effect of the residual systemic inflammation was not readily detectable. Therefore, optimal RA management should aim to achieve not only sustained disease remission but also successful traditional CVD risk reduction, and this may have implications in terms of drug regimen selection.

## Abbreviations

AUC: area under the curve; BMI: body mass index; BP: blood pressure; CAD: coronary artery disease; CV: cardiovascular; CVD: cardiovascular disease; DBP: diastolic blood pressure; DM: diabetes mellitus; DMARD: disease-modifying antirheumatic drug; ESR: erythrocyte sedimentation rate; hs-CRP: high-sensitivity C-reactive protein; IL-6R: interleukin 6 receptor; LDL: low-density lipoprotein; NSAID: nonsteroidal anti-inflammatory drug; RA: rheumatoid arthritis; RF: rheumatoid factor; SBP: systolic blood pressure; TNF: tumour necrosis factor.

## Competing interests

The authors declare that they have no competing interests.

## Authors' contributions

EZ acquired ultrasound data, reevaluated the baseline images offline, analysed the data and drafted the manuscript. AP analysed the data and helped with statistical analysis and drafting the manuscript. KS reevaluated the baseline images offline and analysed the data. KF and CGK were responsible for patient follow-up. KK acquired ultrasound data and reevaluated the baseline images offline. CMP acquired ultrasound data. MM helped in drafting the manuscript. PN was responsible for the statistical analysis. GDK helped with data analysis and interpretation and the drafting of the manuscript. PPS conceived and supervised the study and drafted the manuscript. All authors read and approved the final version of the manuscript.
